# The novel chicken interleukin 26 protein is overexpressed in T cells and induces proinflammatory cytokines

**DOI:** 10.1186/s13567-016-0342-0

**Published:** 2016-06-16

**Authors:** Anh Duc Truong, Boyeong Park, Jihye Ban, Yeong Ho Hong

**Affiliations:** Department of Animal Science and Technology, Chung-Ang University, Anseong, 17546 Republic of Korea

## Abstract

In the present study, we describe the cloning and functional characterization of chicken interleukin 26 (ChIL-26). ChIL-26, a member of the IL-10 cytokine family, induces the production of proinflammatory cytokines by T cells. The ChIL-26 cDNA encodes an 82-amino-acid protein whose amino acid sequence has 22.63, 46.31 and 43.15% homology with human IL-26, pig IL-26 and canary IL-26, respectively. ChIL-26 signals through a heterodimeric receptor complex composed of the IL-20R1 and IL-10R2 chains, which are expressed primarily in the CU91 T cell line as well as CD4^+^ and CD8^+^ T cells. Recombinant ChIL-26 protein induced Th1 cytokines (IL-16 and IFN-γ), Th2 cytokines (IL-4, IL-6 and IL-10), Th17 cytokines (IL-17A, IL-17D, and IL-17F), and chemokine transcripts (mainly CCL3, CCL4, CCL5, CCL20 and CXCL13) in the CU91 T cell line and in CD4^+^ and CD8^+^ T cells, however IL-18 was not expressed in the CU91 T cell line. Taken together, the data demonstrates that T cells express the functional ChIL-26 receptor complex and that ChIL-26 modulates T cell proliferation and proinflammatory gene expression. To the best of our knowledge, this is the first report of cloned ChIL-26. We evaluated its functional roles, particularly in the pathogenic costimulation of T cells, which may be significantly associated with the induction of cytokines.

**Electronic supplementary material:**

The online version of this article (doi:10.1186/s13567-016-0342-0) contains supplementary material, which is available to authorized users.

## Introduction

Interleukin 26 (IL-26) was originally discovered in humans [1] and zebrafish [2]. Human IL-26 (HuIL-26) was cloned as a novel cDNA clone, denoted as AK155, displaying weak but significant sequence homology (approximately 25% identity) to HuIL-10. The genes encoding the ligands of the IL-10 family are located on human chromosome 1 (Chr1) (IL-10, IL-19, IL-20, and IL-24) [3, 4] and Chr12 (IL-22 and -24), and genes for their receptors are located on Chr1 (IL-22R1), Chr3 (IL-20R2), Chr6 (IL-20R1, IL-22BP, and IFNGR1), Chr11 (IL-10R1) and Chr21 (IFNAR2, IL-10R2, IFNAR1 and IFNGR2) [2, 5].

The HuIL-26 gene is located on chromosome 12q15, between the genes for two other important class 2 cytokines, gamma interferon (IFN-γ) and IL-22. IL-26 is often coexpressed with IL-22 by activated T cells, especially Th17 cells [6, 7]. It signals through a heterodimeric receptor complex composed of the IL-20R1 and IL-10R2 chains [8]. HuIL-26 receptors are expressed primarily on non-hematopoietic cell types, particularly epithelial cells [7].

In chickens, only four avian members of the IL-10 family have been identified: IL-10, IL-19, IL-22, and IL-26. Similar to HuIL-26, chicken IL-26 (ChIL-26) is encoded in the same cluster with IL-10 on chromosome 26, in a syntenic region with human Chr1 [9, 10]. In humans, HuIL-26 has been reported to signal via the IL-10R2/IL-20R1 heterodimeric receptor [8, 11]. While IL-10R2 is broadly expressed, IL-20R1 is expressed in many epithelial cell types but not in hematopoietic cells [12, 13]. The only biological activity of IL-26 reported so far is the upregulation of IL-8, IL-10, tumor necrosis factor alpha (TNF-α) and CD54 expression in intestinal epithelial cell lines, in association with STAT3 and/or STAT1 phosphorylation [8, 12].

Recently, HuIL-26 was functionally characterized, and His-HuIL-26 was shown to induce IL-10 and IL-8 in the Colo-205 colon cancer cell line and IL-8 in the Lovo colon cancer and HaCaT cell lines [8]. HuIL-26 induces the production of proinflammatory cytokines and many chemokines (mainly CCL20) in myeloid cells and CD4^+^ T cells [14–16]. IL-26 is also produced by activated T cells and targets epithelial target cells for signal transduction [6, 17]. However, the molecular cloning and functional characterization of ChIL-26 have not yet been performed.

We therefore report here, for the first time, the cloning and functional characterization of ChIL-26. In addition, we examined the biological effects of recombinant ChIL-26 (rChIL-26) protein in the CU91 chicken T cell line, CD4^+^ T cells, and CD8^+^ T cells. We observed increased inflammatory responses, and production of proinflammatory molecules.

## Materials and methods

### Cloning and expression of rChIL-26

To clone full-length ChIL-26, the predicted ChIL-26 coding sequence (GenBank accession # XM_004937561) was amplified from total RNA of the intestinal mucosal layer using the following restriction enzyme-anchored primers: *Eco*RI-anchored forward primer, 5′-CGGAATTCATGAAAAATGTTTTCAGTCATCTTGG-3′; and *Hin*dIII-anchored reverse primer, 5′-CCAAGCTTTACTATGGTTTGGATGTAGGCCT-3′ (restriction sites are underlined). Total RNA was isolated using TRizol reagent (Invitrogen, Carlsbad, CA, USA) as described [18] from the intestinal mucosal layer of White Leghorn chickens, kindly provided by the Animal Biosciences and Biotechnology Laboratory (Beltsville, MD, USA) of the USDA-Agricultural Research Service. The first-strand cDNA was subsequently synthesized using a Maxima First Strand cDNA synthesis kit (Thermo Scientific Inc., Waltham, MA, USA). Polymerase chain reaction (PCR) was performed to amplify the full-length ChIL-26 cDNA, under the following conditions: initial denaturation at 94 °C for 3 min, 35 cycles of denaturation at 94 °C for 30 s, annealing at 60 °C for 30 s, and extension at 72 °C for 30 s and a final extension at 72 °C for 5 min. The newly synthesized full-length ChIL-26 gene was directly inserted into the pCR2.1-TOPO vector (Invitrogen, Carlsbad, CA, USA), followed by transformation into *Escherichia coli* TOP10 (Invitrogen). Transformed *E. coli* TOP10 cells were cultured overnight in Luria–Bertani media (Difco™ and BBL™, NJ, USA) at 37 °C. A transformant was selected by a combination of PCR screening and sequencing (Genotech Inc., Daejeon, Republic of Korea). For cloning into *E. coli* expression vectors, the ChIL-26-expressing plasmid was digested with the endonucleases *Eco*RI (Invitrogen) and *Hin*dIII (Promega, Madison, WI, USA). The digested ChIL-26 fragment was purified from an agarose gel using a QIAQuick gel extraction kit (QIAgen, Valencia, CA, USA) and then ligated into digested pET32a (EMD Millipore, Billerica, MA, USA). To express rChIL-26 in *E. coli*, the purified ChIL-26-pET32a plasmid was introduced into *E. coli* BL21 (Invitrogen).

The expression of rChIL-26 was induced by adding 1 mM IPTG (USB Corporation, Cleveland, OH, USA), and bacteria were cultured at 37 °C overnight at 250 rpm. The bacterial cells were pelleted by centrifugation at 3500 ×* g* for 30 min at 4 °C. The supernatant was removed and the pellet treated with B-PER bacterial protein extraction reagent (Thermo Scientific). Recombinant ChIL-26 was purified using HisPur cobalt resin (Thermo Scientific) in the first step of purification as per the manufacturer’s instructions. To remove the endotoxin contaminants, we combined affinity chromatography with a non-ionic detergent washing step as previously described [19]. The purified protein was concentrated and the buffer changed by ultrafiltration using a 3000-molecular-weight-cutoff membrane (EMD Millipore). The samples were dialyzed in phosphate-buffered saline (PBS; pH 7.2) overnight, using SnakeSkinTM dialysis tubing (Thermo Scientific), with stirring, and were analyzed by SDS-PAGE.

### Antibodies

The following antibodies were used: anti-chicken CD4 (IgG1, k) and anti-chicken CD8 monoclonal antibodies (CT-8), purchased from LifeSpan BioSciences, Inc. (Seattle, WA, USA), anti-His (C-Term)–horseradish peroxidase (HRP) antibody was purchased from Invitrogen and Biotin-labeled goat anti-mouse Ig and Plus-DM streptavidin particles were also purchased from BD Biosciences (San Jose, CA, USA).

### Isolation of CD4^+^, CD8^+^ and splenic T cells

Spleens were obtained from 4 to 5 week-old disease-free Ross 308 chicks (YangJi Hatchery, Pyeongtaek, Republic of Korea). Total cells were strained through a cell strainer (mesh size, 70 µm; Thermo Scientific) into a 50 mL tube. CD4^+^ and CD8^+^ cells were isolated using a BD IMag magnetic bead cell separation system according to the manufacturer’s instructions (BD Biosciences Pharmingen) and described previously [20]. Briefly, the cell concentration was adjusted to 2 × 10^7^ mL^−1^ and 50 µg/mL of anti-CD4 or anti-CD8 monoclonal antibody (LifeSpan BioSciences) was added as determined in preliminary titration experiments. The cells were then incubated on ice for 45 min and washed twice with Hank’s balanced salt solution (HBSS) (Invitrogen) containing 10% fetal bovine serum (FBS). Biotinylated goat anti-mouse IgG (BD Biosciences Pharmingen) was added at the appropriate concentration, and the cells were incubated for 15 min on ice and then washed with an excess volume of 1 × BD IMag buffer (BD Biosciences Pharmingen). Next, 50 µL of streptavidin-labeled magnetic particles (BD IMag Plus-DM streptavidin particles) per 1 × 10^7^ total cells was added, and cells were incubated on ice for 30 min. Magnetic particles were isolated, and the positive and negative cell fractions were suspended in Dulbecco’s modified Eagle’s medium (DMEM) containing 10% FBS.

For stimulation of ChIL-26 and its receptors by concanavalin A (Con A, Sigma), primary T cells were isolated from the spleens of 4 to 5 week-old White Leghorn chickens as previously described [21]. Briefly, splenic T cells were prepared and purified using the Histopaque-1077 (Sigma) density gradient medium by centrifugation at 400 × *g* for 20 min to separation of single cell suspension. Splenic T cells (1 × 10^6^ cells/well) were cultured in 6-well plates (Nunc™, Thermo Scientific) with 5 mL of DMEM (Invitrogen, Carlsbad, CA, USA) containing 100 IU/mL penicillin, 100 mg/mL streptomycin, and 10% heat-inactivated FBS (Invitrogen) in a humidified 5% CO_2_ atmosphere at 37 °C and 5 μg/mL of Con A was added. Cells were harvested after 24, 48 or 72 h of Con A stimulation, and total RNA was extracted from cells as described [18].

### In vitro biological function analysis

The CU91 chicken T cell line [22] was cultured in DMEM (Invitrogen) containing 100 IU/mL penicillin, 100 mg/mL streptomycin, and 10% heat-inactivated FBS (Invitrogen) in a humidified 5% CO_2_ atmosphere at 37 °C. The CU91 T cell line and CD4^+^ and CD8^+^ T cells were cultured at 1 × 10^6^ cells/well in 6-well plates and then treated with either medium alone, lipopolysaccharide (LPS) (5 µg/mL; from *Salmonella enterica* serotype Typhimurium; (Sigma), rChIL-26 (200 ng/mL) or LPS/rChIL-26). Cells were collected at 24, 48 and 72 h post treatment. In pilot experiments, rChIL-26 demonstrated a significantly greater effect at a concentration of around 200 ng/mL for the activation of certain kinases. After incubation, the cells were collected and total RNA was extracted from cells using TRizol reagent (Invitrogen) and cDNA was synthesized as described [18].

### Western blotting

**Figure 1:**
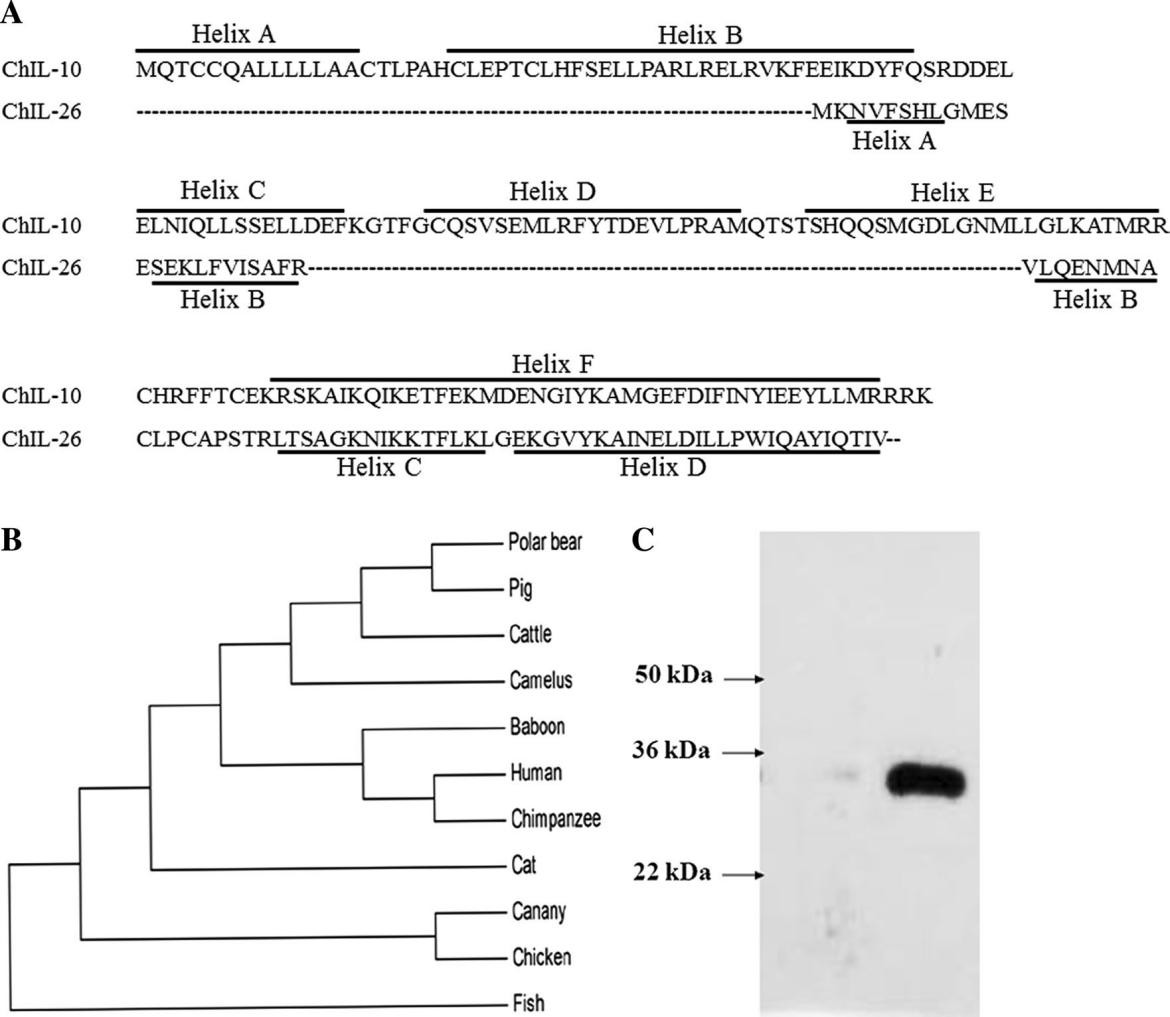
**Sequence comparison, phylogenetic tree of amino acid and western blot analysis of ChIL-26.** **A** Chicken IL-10 (Accession # NP 001004414) and ChIL-26 amino acid sequences were aligned and secondary helices of IL-10 and IL-26 were shown in vertical and horizontal lined box, respectively. **B** Phylogenetic tree was constructed by Phylodendron program using amino acid sequences aligned with BioEdit and Mega4 and (C) Western blot analysis of ChIL-26 recombinant protein. *E. coli* BL21(DE3) transformed with the pET32a(+)-ChIL-26 plasmid was induced with 1.0 mM IPTG, purified with a HisPur Cobalt resin and analyzed by Western blotting using anti-His (C-Term)-HRP Antibody.

The ChIL-26 recombinant protein (100 ng/µL) was separated by SDS-PAGE and then transferred to a polyvinylidene difluoride (PVDF) membrane (Invitrogen) using an iBlot^®^ gel transfer device (Invitrogen). The transferred PVDF membrane was blocked with 5% nonfat milk (Sigma) dissolved in PBS containing 0.05% Tween 20 (PBST). The membrane was incubated with anti-His (C-Term)–horseradish peroxidase (HRP) antibody overnight at 4 °C, washed three times with PBST. Detection of immunoreactive bands was performed using Western Lightning Plus-ECL substrate (Thermo Scientific) as per the supplier’s instructions. A single ChIL-26 protein band with an apparent molecular mass of approximately 33.0 kDa was observed (Figure 1C). The increased size over the predicted 10.9 kDa was due to the three epitope tags (polyhistidine, S-protein, and thioredoxin) in the recombinant protein.

### Quantitative real-time PCR (qRT-PCR) analysis of cytokine transcripts

For analysis of mRNA transcripts, 2 µg of total RNA isolated from cells was treated with DNase I (Sigma) according to the manufacturer’s instructions to eliminate possible genomic DNA contamination. Briefly, 2 µg of total RNA was treated with 1.0 unit of DNase I in 1.0 μL of 10× reaction buffer (Thermo Scientific) and incubated for 30 min at 37 °C. Total RNA was reverse transcribed using a Maxima First Strand cDNA synthesis kit (Thermo Scientific) according to the manufacturer’s recommendations. Briefly, 2 μg of RNA was combined with 0.2 μg of oligo (dT)_18_ primer and RNase-free water for a total volume of 12.0 μL. Next, 4.0 μL of 5× reaction buffer, 20 units of Ribolock RNase inhibitor, 10 mM dNTP mix, and 200 units of RevertAcid M-MuLV reverse transcriptase were added. The mixture was incubated at 42 °C for 60 min and then heated at 70 °C for 5 min to terminate the reaction. To analyze the transcripts of various cytokines, cytokine primers were designed using Lasergene software (DNASTAR Inc., Madison, WI, USA) (Table 1). Quantitative real-time PCR (qRT-PCR) was performed with 2× Power SYBR Green master mix (Roche, IN, USA) according to the manufacturer’s instructions, using a LightCycler 96 system (Roche). In brief, cDNA (100 ng) was added to a reaction mixture of 10 μL 2× Power SYBR Green master mix (Roche), 0.5 μL of each primer (10 pmol/µL) and RNase-free water for a total volume of 20 μL. GAPDH was used as an internal control to normalize the quantification of target genes. The relative quantification of gene-specific expression was calculated using the 2^−ΔΔCt^ method after normalization with chicken GAPDH [23]. All qRT-PCR reactions were performed in triplicate.

**Table 1:** **Primer sequences for real-time PCR analyses of cytokine transcript expression**

Primer	F/R	Nucleotide sequencing (5′-3′)	Accession no
GAPDH	Forward	TGC TGC CCA GAA CAT CAT CC	NM_204305
	Reverse	ACG GCA GGT CAG GTC AAC AA	
CCL3	Forward	CAT TGC CTC CGC CTA CAT	EU999777
	Reverse	ACT CCT CGG GGT TTA CAC ATA	
CCL4	Forward	CCC CTT GTC ATC GGT CAC	NM_204720
	Reverse	AGA GGC AGG AGC AGA GCA	
CCL5	Forward	CAG CAA ATG CCC ACA GG	NM_001045832
	Reverse	TGC AGC TCC AGG AAG TTG AT	
CCL20	Forward	AGG CAG CGA AGG AGC AC	NM_204438
	Reverse	GCA GAG AAG CCA AAA TCA AAC	
CXCL13	Forward	GCC TGT GCC TGG TGC TC	XM_420474.4
	Reverse	TGC CCC CTT CCC CTA AC	
IFN-γ	Forward	AGC TGA CGA CGG TGG ACC TAT TAT T	HQ739082
	Reverse	GGC TTT GCG CTG GAT TC	
IL-4	Forward	AGC ACT GCC ACA AGA ACC TG	NM_001007079
	Reverse	CCT GCT GCC GTG GGA CAT	
IL-6	Forward	CAA GGT GAC GGA GGA GGA C	JQ897539
	Reverse	TGG CGA GGA GGG ATT TCT	
IL-8 (CXCLi2)	Forward	GGC TTG CTA GGG GAA ATG A	NM_205498
	Reverse	AGC TGA CTC TGA CTA GGA AAC TGC	
IL-10	Forward	CTG TCA CCG CTT CTT CAC CT	AJ621254
	Reverse	ACT CCC CCA TGG CTT TGT A	
IL-16	Forward	TGC CTC ACA AGA ATC AAC AAC T	AB104417.2
	Reverse	ATA GAG CCC TTC CCA CCT TC	
IL-17A	Forward	TGT CTC CGA TCC CTT GTT CT	AM773756
	Reverse	GTC CTG GCC GTA TCA CCT T	
IL-17D	Forward	ACC CCA CAA GAT ACC CTA AAT AC	EF570583
	Reverse	GTG CTG CGG AAG TGA AAA T	
IL-17F	Forward	CTC CGA TCC CTT ATT CTC CTC	NM_204460
	Reverse	GTC CTG GCC GTA TCA CCT T	
IL-18	Forward	ATT TTC CCA TGC TCT TTC TCA	HM854281
	Reverse	GGA ATG CGA TGC CTT TTG	
IL-26	Forward	AAT GCC TGT CTT CCG TGT G	XM_004937561
	Reverse	TCA TTG ATG GCC TTG TAG ACC	
TNFSF15	Forward	CCT GAG TAT TCC AGC AAC GCA	NM_001024578
	Reverse	ATC CAC CAG CTT GAT GTC ATC AAC	
IL-10R2	Forward	GAG CAG ACC ACC CAT AAC G	NM_204857
	Reverse	ATA CCA AAA GGC AAA GAA ACA A	
IL-20R1	Forward	GTG GAT GAA TTG CTG GGT AAG	XM_419723
	Reverse	TCT GTG ATG CCG TGT GCT AT	

### Bioinformatics and statistical analyses

Multiple-sequence alignments were analyzed using Lasergene software (DNASTAR Inc., Madison, WI, USA), and a phylogenetic tree was constructed using the neighbor-joining method, with a bootstrap value of 1000, in the MEGA4 program [24]. Signal peptides and N-like glycosylation were predicted using NetNGlyc software [25]. Statistical analysis was performed using IBM SPSS software (SPSS 21.0 for Windows; SPSS, Chicago, IL, USA). The results are expressed as mean ± standard error of mean (SEM) at three independent experiments for each group (*n* = 3) and were compared between groups using the Duncan’s multicomparison method.

## Results

### Chicken IL-26

ChIL-26 consists of 82 amino acids and has a predicted isoelectric point of 9.6 and a molecular mass of 10.9 kDa (Additional file 1). In contrast to HuIL-26, ChIL-26 does not contain signal peptides. Moreover, HuIL-26 has two glycosylation sites (NFIL and NKGI sites) and ChIL-26 has three glycosylation sites (NVFS, NACL and NELD sites). The ChIL-26 protein has 43.15% and 46.31% amino acid similarities to canary and pig IL-26, respectively, whereas the similarities to other mammalian IL-26 proteins are approximately 23% (Table 2). Analysis of the secondary structure showed four highly conserved helix structures between ChIL-10 and ChIL-26 (Figure 1A). A phylogenetic tree of predicted IL-26 proteins extracted from GenBank is shown in Figure 1B. The ChIL-26 protein comprises a grouping distinct from those of mammalian and amphibian species (Figure 1B).

**Table 2:** **Chicken IL-26 amino acid similarity and identity with other species**

	Chicken		Accession no
	Similarity (%)	Identity (%)	
Human	27.36	22.63	NP_060872
Fish	22.1	20	NP_001018635
Baboon	27.89	22.63	XP_003906792
Canary	46.84	43.15	XP_009093343
Chimpanzee	27.36	22.63	XP_003808754
Camelus	23.68	21.57	AIL25449
Polar bear	28.42	23.15	XP_008682482
Cat	27.89	22.63	XP_006933945
Cattle	28.42	23.15	XP_005909110
Pig	48.94	46.31	ABJ96481

The amino acid similarity and identity were analyzed using BLASTP with a BLOSUM62 scoring matrix.

### ChIL-26 receptor complex in T cells

**Figure 2:**
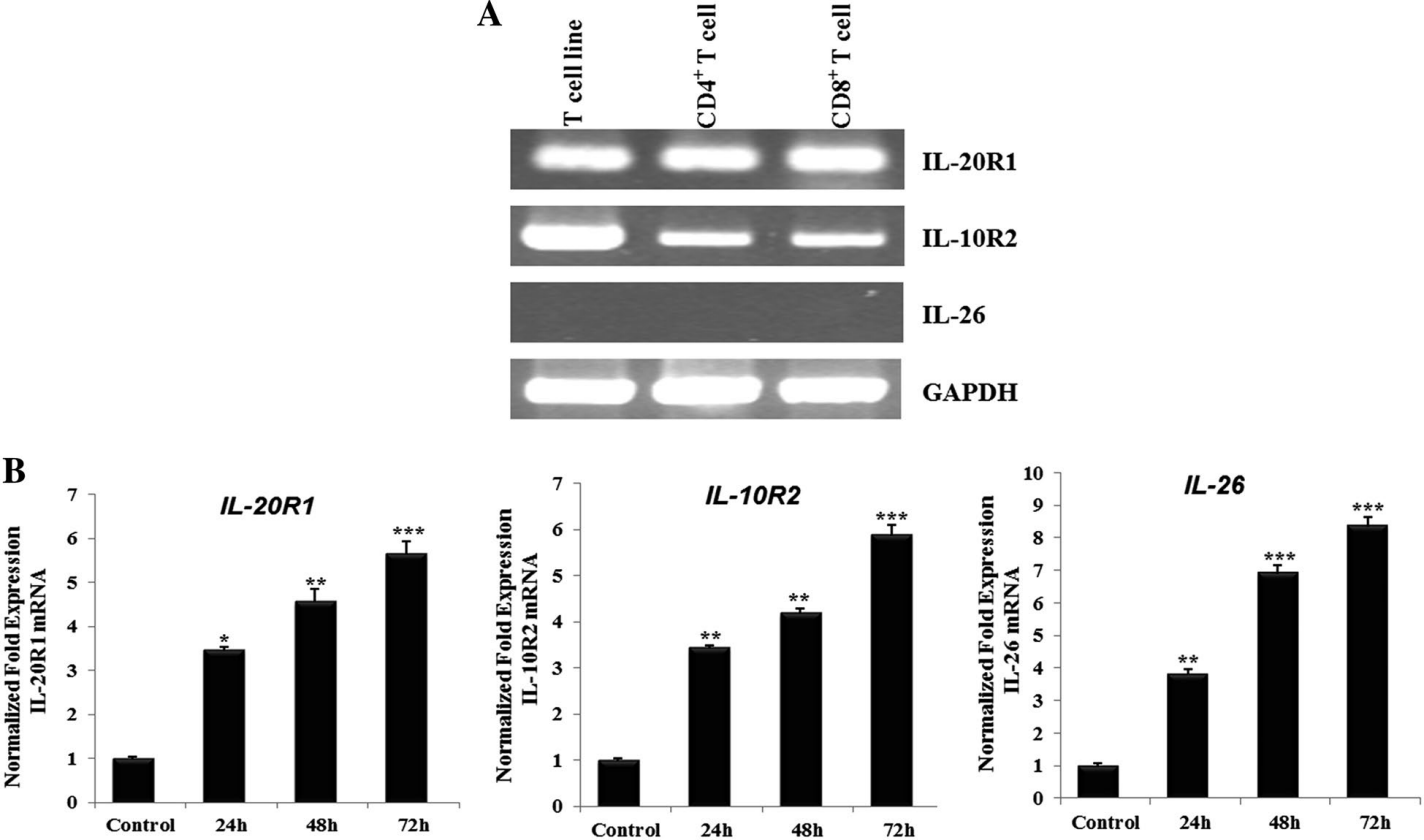
**Receptors and GAPDH are expressed but not IL-26 are expressed in T cell.** **A** IL-10 receptor 2 and IL-20 receptor 1 are expressed, but IL-26 is not expressed in T cell (T cell line, CD4^+^ T cell and CD8^+^ T cell). **B** IL-10R2, IL-20R1 and IL-26 expression in primary T cell induced by Con A (5 µg/mL), respectively. The expression of IL-10R2, IL-20R1 and IL-26 mRNA as analyzed by qRT-PCR analysis of mRNA derived from T cell as indicated. Data are presented as the mean ± SEM (*n* = 3) of three independent experiments: *p < 0.05, **p < 0.01, and ***p < 0.001.

To determine whether ChIL-26 and the ChIL-26 receptor complex, consisting of IL-10R2 and IL-20R1, are expressed in T cells and to study this ligand-receptor system, we analyzed ChIL-26, IL-10R2 and IL-20R1 mRNA expression in the chicken CU91 T cell line and in CD4^+^ and CD8^+^ T cells. RT-PCR was performed to confirm IL-10R2 and IL-20R1 mRNA expression and demonstrated that the ChIL-10R2 and ChIL-20R1 genes were expressed and that ChIL-26 was not expressed in all T cells tested. A low expression of IL-10R2 in CD4^+^ and CD8^+^ T cell compared to CU91 T cell line may be caused by non-treatment of Con A because CU91 T cell line established using interleukin-containing conditioned medium or concanavalin A stimulated T cells (Figure 2A). Moreover, Con A treatment had an effect on ChIL-26, IL-10R2 and IL-20R1 mRNA expression in primary T cells (Figure 2B). IL-10R2 mRNA levels increased 3.4-, 4.19- and 5.9-fold compared with the control group after Con A treatment for 24, 48 (*p* < 0.01), and 72 h (*p* < 0.001), respectively. IL-20R1 mRNA levels also significantly increased (3.5-, 4.6- and 5.6-fold) compared with the control group, with varying levels of significance (Figure 2B). In addition, ChIL-26 expression increased considerably (3.8-, 6.9- and 8.4-fold) compared with the control group, also with varying levels of significance (Figure 2B). Therefore, mRNA expression of ChIL-26 and its receptors, IL-10R2 and IL-20R1, was affected by Con A treatment of splenocytes.

**Figure 3:**
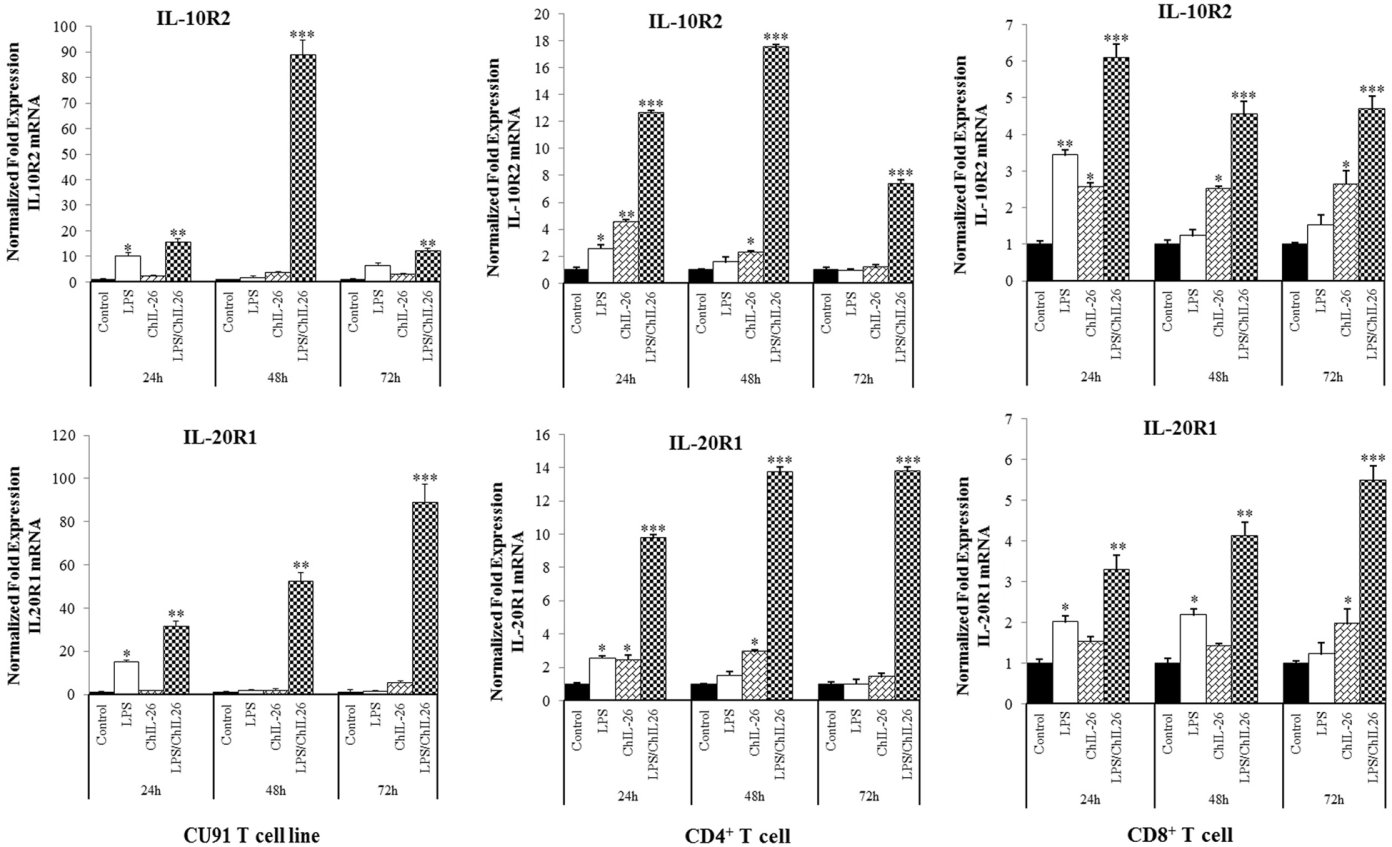
**Interleukin 20 receptor 1 (IL-20R1) and interleukin 10 receptor 2 (IL-10R2) expressed in T cell.** IL-20R1 and IL-10R2 mRNA expressed in CU91 T cell line, CD4^+^ T cell and CD8^+^ T cell as demonstrated by quantitative RT-PCR. Data are presented as the mean ± SEM (*n* = 3) of three independent experiments: *p < 0.05, **p < 0.01, and ***p < 0.001.

Moreover, the expression of IL-10R2 and IL-20R1 mRNAs was confirmed in the CU91 T cell line and in CD4^+^ and CD8^+^ cells by qRT-PCR. The results indicated that the expression of IL-10R2 and IL-20R1 genes was upregulated in all T cells, particularly those cotreated with LPS and ChIL-26, including the CU91 T cell line, with 89.0-fold upregulation after 48 h and 88.9-fold upregulation after 72 h of treatment (Figure 3). Similar to the results obtained for the CU91 T cell line, IL-10R2 and IL-20R1 mRNA expression in CD4^+^ and CD8^+^ T cells was also highly upregulated by LPS/ChIL-26 cotreatment. In CD4^+^ T cells, IL-10R2 and IL-20R1 were upregulated 17.5- and 13.8-fold after 48 h treatment, and these receptors were increased 6.1-fold after 24 h and 5.5-fold after 72 h treatment with LPS/ChIL-26 in CD8^+^ T cells (Figure 3). This observation suggested that IL-10R2 and IL-20R1 are expressed on T cells and may be two chain for the chicken IL-26 receptor complex.

### Costimulation with LPS/ChIL-26 leads to proinflammatory cytokine and chemokine expression in T cells

**Figure 4:**
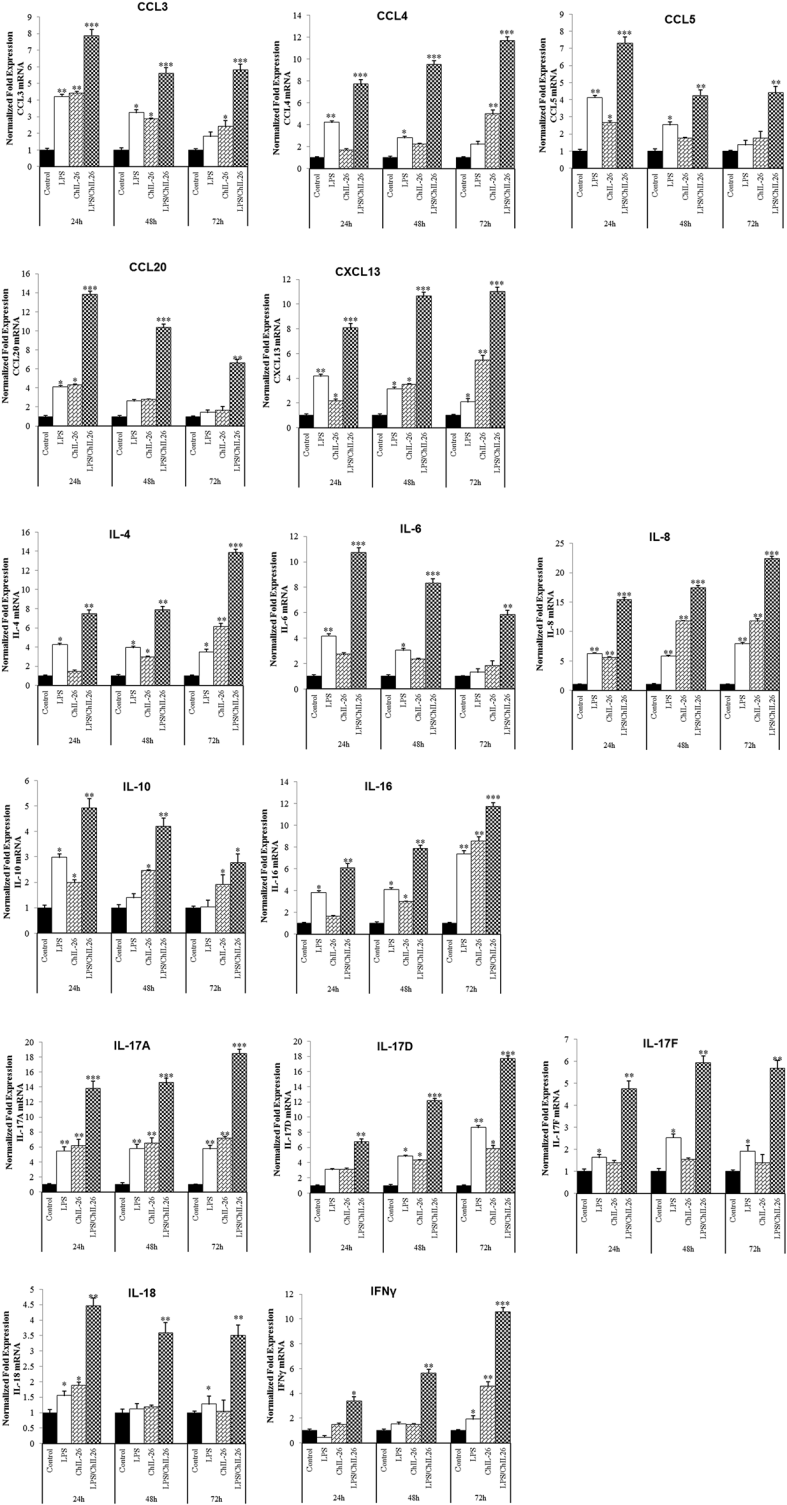
**ChIL-26/LPS induces cytokine secretion by CD8** ^**+**^ **T cells.** The CD8^+^ T cell line was cultured in the presence or absence of 200 ng/mL ChIL-26, with or without 5 µg/mL LPS, and cytokine expression was analyzed by qRT-PCR. The results are indicated as fold increases of mRNA expression compared to that in unstimulated control cells. Data are presented as the mean ± SEM (*n* = 3) of three independent experiments: *p < 0.05, **p < 0.01, and ***p < 0.001.

Previous reports demonstrated that HuIL-26 induces Th1 and Th17 as well as Th2 cytokines [14, 26]; therefore, we examined the transcriptional targets of this cytokine. The role of ChIL-26 in the transcriptional regulation of Th17 cytokines involved the induction of IL-17A, IL-17D and IL-17F cytokines. LPS and ChIL-26 treatment significantly enhanced the expression of IL-17A and IL-17D in all T cells at 72 h. In addition, IL-17F expression was also increased in the CU91 T cell line and in CD4^+^ and CD8^+^ cells at all time points, when cells were treated with LPS/ChIL-26 (Figure 4; Additional files 2 and 3). The IL-26-regulated expression of IL-17A, IL-17D and IL-17F by T cells and particularly in CU91 T cell line.

The levels of the IL-4, IL-6, and IL-10 cytokines, which correspond to Th2 cytokines in birds, significantly increased following stimulation with LPS and ChIL-26 compared to the control. IL-4 mRNA was highly increased at 72 h, and its levels maximized at 131-, 16.4- and 13.8-fold in the CU91 T cell line and in CD4^+^ and CD8^+^ T cells, respectively compared to the control (*p* < 0.001) (Figure 4; Additional files 2 and 3). The expression of IL-6 and IL-10 mRNAs also increased significantly at 24 h post-LPS/ChIL-26 stimulation compared to the control in all T cells (*p* < 0.001) (Figure 4; Additional files 2 and 3).

IL-18 mRNA was not increased in the CU91 T cell line stimulated with LPS or ChIL-26, or both, however it increased 4.4-fold with LPS and ChIL-26 or with ChIL-26 only in CD4^+^ and CD8^+^ T cells (p < 0.05) (Figure 4; Additional files 2 and 3). The expression of IFN-γ, a Th1 cytokine, also increased, and reached its maximum expression level after 72 h of costimulation with LPS and ChIL-26 in all T cells (*p* < 0.01) (Figure 4; Additional files 2 and 3).

IL-16 mRNA was also highly expressed 24 h after costimulation in the CU91 T cell line and in CD4^+^ T cells and at 72 h in CD8^+^ T cells (*p* < 0.01) (Figure 4; Additional files 2 and 3). The LPS and ChIL-26 combination also had a positive effect on chemokine expression in T cells. When LPS and ChIL-26 were used in combination, the expression of CCL3, CCL4, CCL20, and CXCL13 mRNAs increased significantly in the CU91 T cell line at 24 h, in CD4^+^ cells at 24 h (CCL4 and CCL20) and 48 h (CXCL13), and CD8^+^ cells at 24 h (CCL3 and CCL20) and 72 h (CCL4 and CXCL13) compared with controls (*p* < 0.001) (Figure 4; Additional files 2 and 3). The expression of CCL5 and IL-8 (CXCLi2) mRNAs increased significantly compared with controls at 48 h (*p* < 0.001) (Additional files 2). In CD4^+^ and CD8^+^ T cells, the highest expression levels of all chemokines measured over all time points were seen with LPS and ChIL-26 treatments. Collectively, these results demonstrated that ChIL-26 upregulates the expression of proinflammatory cytokines and chemokines mRNA in the CU91 T cell line and in CD4^+^ and CD8^+^ T cells.

## Discussion

This is the first report demonstrating a role for ChIL-26 in chickens. ChIL-26 is a novel member of the IL-10-like cytokine family and shares the IL-10R2 subunit with IL-10 [27] and the IL-20R1 subunit with IL-20 [28]. The IL-26 gene and its corresponding protein also share some sequence homology with HuIL-10 and ChIL-10. IL-10 has anti-inflammatory properties, however we demonstrated here that IL-26 has proinflammatory functions in T cells and induces cytokine upregulation upon costimulation with LPS. Other research groups first identified the receptors for HuIL-26 [8, 11]. HuIL-26 signals through a heterodimeric receptor complex composed of IL-20R1 and IL-10R2 chains. IL-20R1 functions as the specific ligand binding chain for IL-26, and IL-10R2 functions as an essential second chain to complete assembly of the active receptor complex. Similar to humans, signaling via chicken IL-10R2 and IL20R1 is also activated by LPS and ChIL-26 costimulation (Figure 3). Neutralizing antibodies against either the IL-20R1 or IL-10R2 chain can block induction of HuIL-26 signaling [8, 11]. In contrast to the anti-inflammatory properties of IL-10, we demonstrated that ChIL-26 modulates the proinflammatory functions of T cells. Similar to HuIL-26, ChIL-26 upregulates the gene expression of proinflammatory cytokines in T cells.

Depending on the T cell source, cytokines have been differentiated into Th1, Th2 and Th17 cytokines. Previous studies demonstrated an induction of HuIL-26 production particularly in Th1 cells [14]. Crohn’s disease is considered to represent Th1- and Th17-mediated intestinal inflammation, while ulcerative colitis has features, at least to some degree, of Th2-mediated colitis [29–31]. Our studies demonstrated that ChIL-26 strongly induces Th1 cytokines (IL-16 and IFN-γ; but with an absence of IL-18 in the CU91 T cell line), Th2 cytokines (IL-4, -6 and -10), Th17 cytokines (IL-17A, IL-17D and IL-17F), and chemokines (mainly CCL3, CCL4, CCL5, CCL20, CXCLi2 and CXCL13) in T cells (Figure 4; Additional files 2 and 3). Overall, the cytokine and chemokine genes were expressed more highly in the CU91 T cell line than in CD4^+^ and CD8^+^ T cells upon costimulation with LPS and ChIL-26. Researcher groups previously reported that resting T cells expressed lager diversity of genes and the patterns of gene expression showed markedly downregulation than transformed T cells cause by the different pathway involving antigen-presenting cell [32–34]. Therefore, the differences in gene expression for the CU91 T cell line and CD4^+^ and CD8^+^ T cells may result from disparities in the efficiency of antigen presentation via the MHC class I and II pathways, in the context of LPS and ChIL-26 cotreatment [35–37].

Avian IL-26 is another member of the IL-10 family, and chicken T cells express a functional IL-26 receptor complex (IL10R2 and IL-20R1). LPS binds to Toll-like receptor 4 (TLR4) on the cell surface and forms the LPS-TLR4 complex, which triggers both MyD88-dependent and MyD88-independent TRIF-dependent pathways. Toll-like receptor (TLR) family including TLR-4 expressed in chicken immune T cell subsets (CD4^+^ and CD8^+^ T cell) and cell lines (B and T cell lines) [38]. Signaling through the MyD88-dependent pathway is responsible for early-phase NF-κB and MAPK activation, which facilitates the induction of proinflammatory cytokines, chemokines, adhesion molecules, and MHC or costimulatory molecules [39]. As expected, the cytokines in T cells were significantly more highly expressed in cells receiving LPS/ChIL-26 costimulation than in cells treated with either LPS or ChIL-26 alone.

Here, we demonstrated the successful cloning and expression of ChIL-26 and its molecular functions in immune responses in the CU91 chicken T cell line and CD4^+^ and CD8^+^ T cells. In this study, the use of ChIL-26-deficient T cells revealed that ChIL-26 expression is necessary for optimal LPS induction of several cytokines. Therefore, we suggest that ChIL-26 as well as pathogens may influence the expression of Th1, Th2 and Th17 cytokines. Further investigation into the regulation of ChIL-26 gene expression with signaling pathway will undoubtedly reveal new insights into the regulation of T cell proinflammatory gene programs.

## Additional files

10.1186/s13567-016-0342-0 **Nucleotide and deduced amino acid sequences of ChIL-26.** The primers used to amplify the full-length ChIL-26 sequence are underlined.
10.1186/s13567-016-0342-0 **ChIL-26/LPS induces cytokine secretion by the CU91 T cell line.** The CU91 chicken T cell line was cultured in the presence or absence of 200 ng/mL ChIL-26, with or without 5 µg/mL LPS, and cytokine expression was analyzed by qRT-PCR. The results are indicated as fold increases of mRNA expression compared to that in unstimulated control cells. Data are presented as the mean ± SEM (*n* = 3) of three independent experiments: * *p* < 0.05, ** *p* < 0.01, and *** *p* < 0.001.
10.1186/s13567-016-0342-0 **The CD4+ T cell line was cultured in the presence or absence of 200 ng/mL ChIL-26, with or without 5 µg/mL LPS, and cytokine expression was analyzed by qRT-PCR. **The results are indicated as fold increases of mRNA expression compared to that in unstimulated control cells. Data are presented as the mean ± SEM (*n* = 3) of three independent experiments: * *p* < 0.05, ** *p* < 0.01, and *** *p* < 0.001.

## Abbreviations

MHC: major histocompatibility complex
TCR: T cell receptor
CTLs: cytotoxic T lymphocytes
NKs: natural killer cells
IL: interleukin
TNF: tumor necrosis factor
RT: reverse transcription
IPTG: isopropyl β-d-1-thiogalactopyranoside
MAPK: mitogen-activated protein kinase
TLR: toll-like receptor
