# Supplementary figures and images for: The novel chicken interleukin 26 protein is overexpressed in T cells and induces proinflammatory cytokines
Source: Vet Res. 2016 Jun 16;47:65. doi: 10.1186/s13567-016-0342-0 (PMC4911681; doi:10.1186/s13567-016-0342-0)

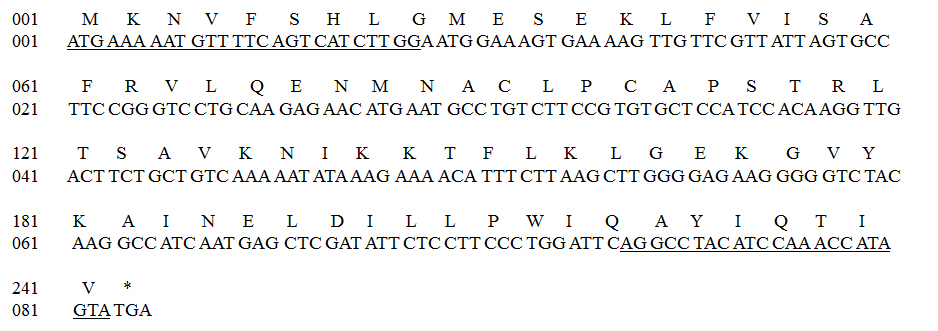

Supplement: Supplementary file 1 — 10.1186/s13567-016-0342-0 Nucleotide and deduced amino acid sequences of ChIL-26. The primers used to amplify the full-length ChIL-26 sequence are underlined. [file 13567_2016_342_MOESM1_ESM.jpg]

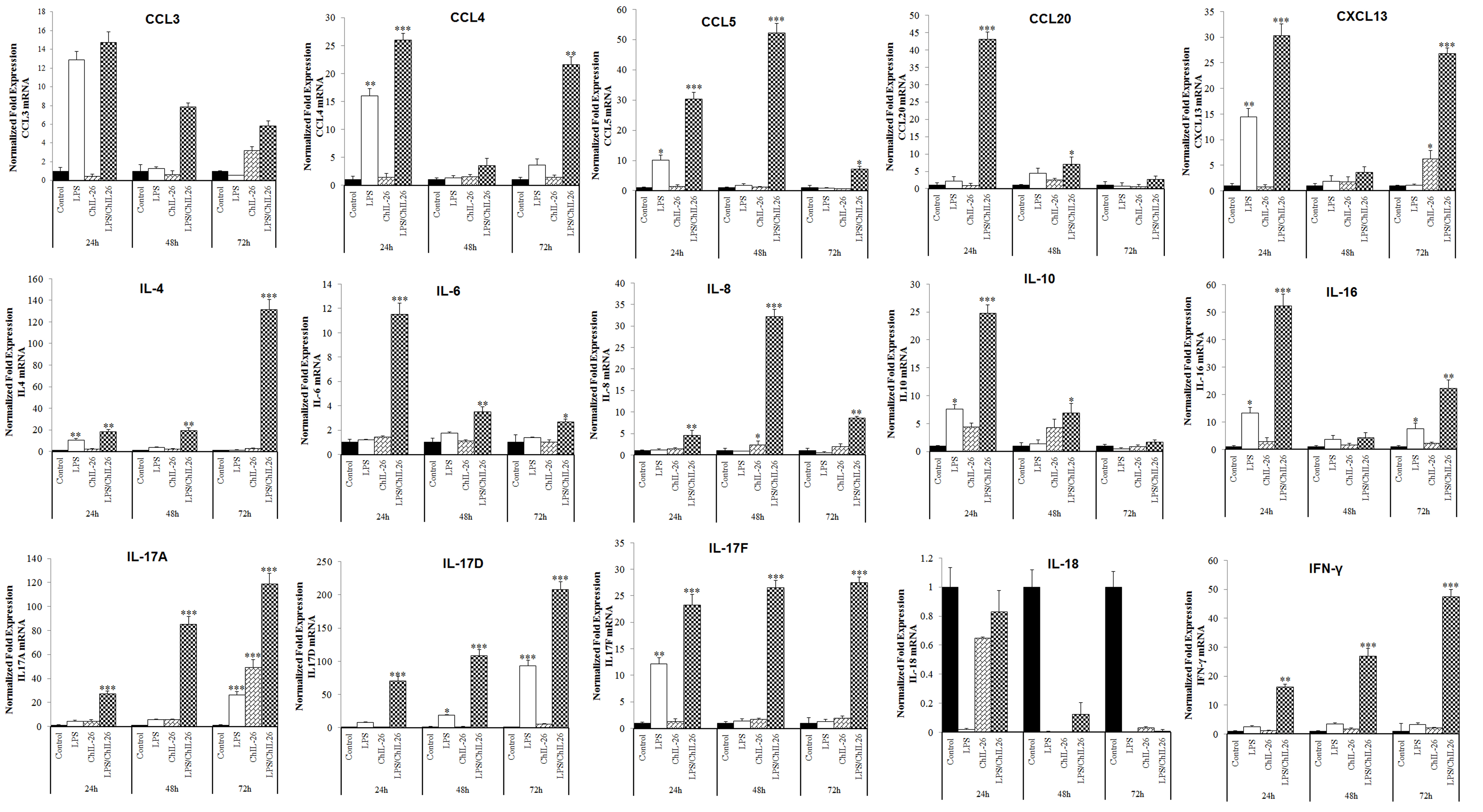

Supplement: Supplementary file 2 — 10.1186/s13567-016-0342-0 ChIL-26/LPS induces cytokine secretion by the CU91 T cell line. The CU91 chicken T cell line was cultured in the presence or absence of 200 ng/mL ChIL-26, with or without 5 µg/mL LPS, and cytokine expression was analyzed by qRT-PCR. The results are indicated as fold increases of mRNA expression compared to that in unstimulated control cells. Data are presented as the mean ± SEM (n = 3) of three independent experiments: * p < 0.05, ** p < 0.01, and *** p < 0.001. [file 13567_2016_342_MOESM2_ESM.jpg]

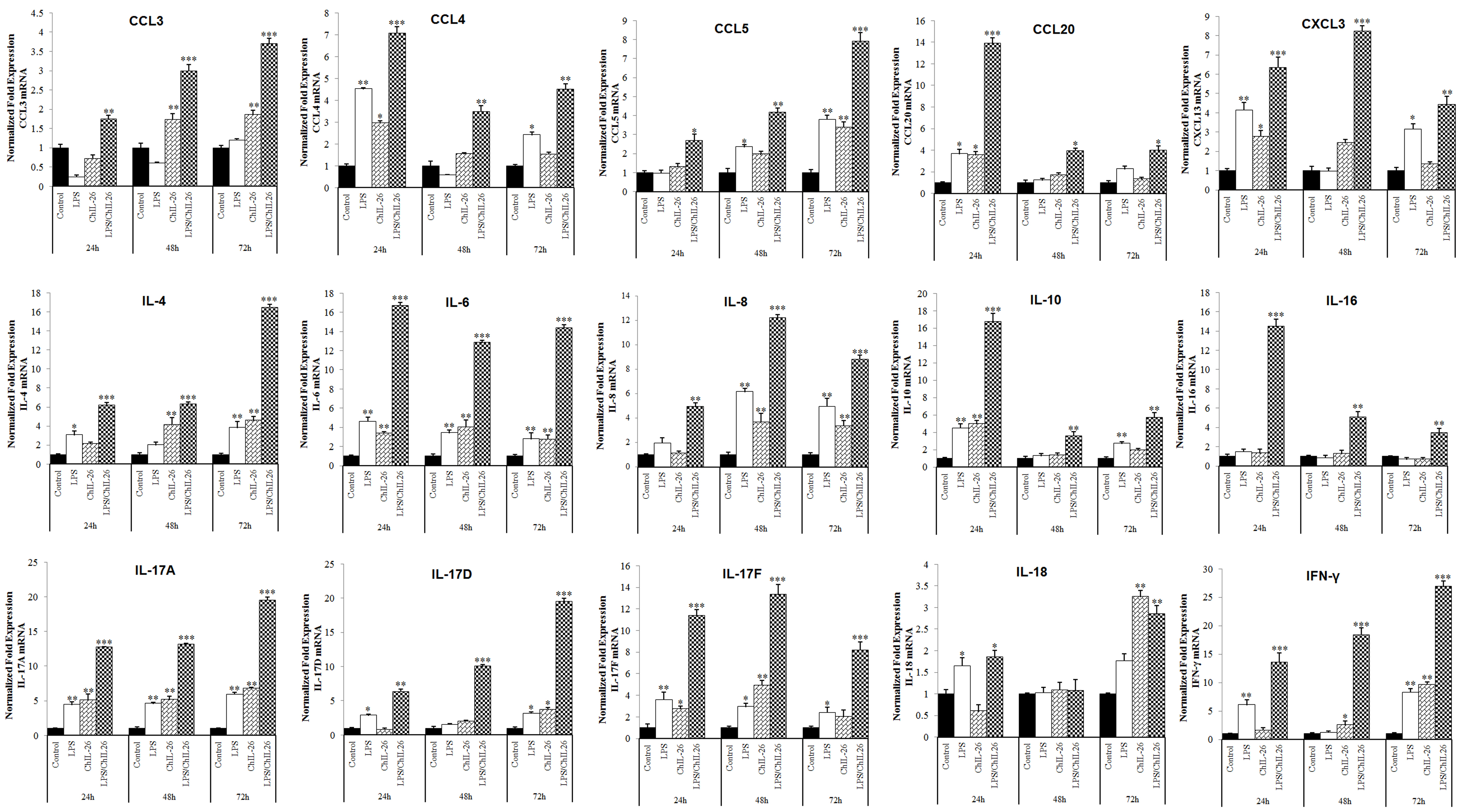

Supplement: Supplementary file 3 — 10.1186/s13567-016-0342-0 The CD4+ T cell line was cultured in the presence or absence of 200 ng/mL ChIL-26, with or without 5 µg/mL LPS, and cytokine expression was analyzed by qRT-PCR. The results are indicated as fold increases of mRNA expression compared to that in unstimulated control cells. Data are presented as the mean ± SEM (n = 3) of three independent experiments: * p < 0.05, ** p < 0.01, and *** p < 0.001. [file 13567_2016_342_MOESM3_ESM.jpg]
